# Disease and the Extended Phenotype: Parasites Control Host Performance and Survival through Induced Changes in Body Plan

**DOI:** 10.1371/journal.pone.0020193

**Published:** 2011-05-25

**Authors:** Brett A. Goodman, Pieter T. J. Johnson

**Affiliations:** 1 Ecology and Evolutionary Biology, University of Colorado, Boulder, Colorado, United States of America; 2 School of Earth and Environmental Sciences, University of Adelaide, Adelaide, South Australia, Australia; Institute of Marine Research, Norway

## Abstract

**Background:**

By definition, parasites harm their hosts. However, some forms of parasite-induced alterations increase parasite transmission between hosts, such that manipulated hosts can be considered extensions of the parasite's phenotype. While well accepted in principle, surprisingly few studies have quantified how parasite manipulations alter host performance and survival under field and laboratory conditions.

**Methodology/Principal Findings:**

By interfering with limb development, the trematode *Ribeiroia ondatrae* causes particularly severe morphological alterations within amphibian hosts that provide an ideal system to evaluate parasite-induced changes in phenotype. Here, we coupled laboratory performance trials with a capture-mark-recapture study of 1388 Pacific chorus frogs (*Pseudacris regilla*) to quantify the effects of parasite-induced malformations on host locomotion, foraging, and survival. Malformations, which affected ∼50% of metamorphosing frogs in nature, caused dramatic reductions in all measures of organismal function. Malformed frogs exhibited significantly shorter jumping distances (41% reduction), slower swimming speeds (37% reduction), reduced endurance (66% reduction), and lower foraging success relative to infected hosts without malformations. Furthermore, while normal and malformed individuals had comparable survival within predator-free exclosures, deformed frogs in natural populations had 22% lower biweekly survival than normal frogs and rarely recruited to the adult population over a two-year period.

**Conclusions/Significance:**

Our results highlight the ability of parasites to deeply alter multiple dimensions of host phenotype with important consequences for performance and survival. These patterns were best explained by malformation status, rather than infection per se, helping to decouple the direct and indirect effects of parasitism on host fitness.

## Introduction

Although typically hidden from view, parasites and pathogens are a ubiquitous component of almost all communities [1]–[2]. Most organisms are infected by many different parasite species, some of which have strong effects on host growth, reproduction, foraging success, behavior, and even elemental stoichiometry [3]–[7]. In some instances, parasite-induced changes help to increase parasite growth, persistence within a host, or transmission between hosts [8]–[11]. Indeed, parasite-induced manipulations of host phenotypes have become one of the most commonly cited illustrations of the ‘extended phenotype’, in which heritable effects of organisms' genes transcend the level of individuals or populations to affect other species in the community [2], [11]–[14]. The premise of this idea is encapsulated within the notion that host behavior and morphology, like other aspects of the phenotype, can be co-opted by a parasite and used to benefit the parasite's genes [15]. For instance, when infected with a nematode, the abdomen of the giant turtle ant (*Cephalotes atratus*) changes in color from black to a rich red, thereby resembling the ripe fruit consumed by frugivorous birds, which function as the parasite's final host [16]–[17]. Because parasite-manipulated hosts may differ in behavior, habitat use, and even reproductive capacity, parasites can effectively create the existence of ‘cryptic subgroups’ that function largely as separate species [2], [5], [18].

While well accepted in principle, surprisingly few studies have quantified how parasite-induced changes in phenotype affect hosts under natural conditions [19]–[21]. Because parasite manipulations can be multidimensional [22], such that more than one aspect of a host's phenotype is altered by infection, a persistent challenge in the study of parasite manipulations involves isolating exactly which dimensions of host function are affected by infection. Furthermore, intrinsic host behaviors (e.g., promiscuity or activity) can influence the likelihood an individual becomes infected in the first place, confounding identification of which behaviors are a cause or consequence of infection [23]. While experiments can help to offset this problem [24]–[25], the use of fixed levels of parasite exposure (e.g., infected and manipulated vs. uninfected and unmanipulated) tends to mask whether observed changes in host performance are due to the specific phenotype under scrutiny or other, unmeasured effects of infection. These obstacles underscore the (1) importance of integrative approaches that combine laboratory and field studies to understand the effects of parasitism on host phenotype and (2) value of systems that provide an opportunity to decouple and independently quantify the effects of infection and phenotypic changes on host performance.

An intriguing class of infections ideally suited to address such challenges involves parasites that induce morphological rather than behavioral changes in their hosts. While many examples of host phenotypic change involve subtle alterations in host behavior, color, or size [11], some parasites cause spectacular changes in host morphology for which the causal relationship is readily apparent. In amphibians, for example, infection by the trematode *Ribeiroia ondatrae* causes dramatic morphological changes in host body plan, such as missing, misshapen or extra limbs [26]–[28]. This extreme phenotypic manipulation, which can affect >50% of emerging frogs in a population, is hypothesized to increase parasite transmission by enhancing bird and mammal predation on infected frogs [29]–[31]. Because of variation in the timing of infections in amphibian larvae [32]–[33], individual hosts within a pond exhibit considerable variation in both infection and malformation status [27], [34], thus providing an ideal system in which to disentangle the effects of infection and pathology among naturally occurring hosts. Moreover, given that malformations affect performance of metamorphic frogs but infection occurs only during larval development, parasite-induced changes are unlikely to influence the accumulation of additional parasites prior to metamorphosis, which can confound a clear understanding of the causal relationship between infection and phenotype.

Here, we used the *Ribeiroia*-amphibian system to broadly evaluate the consequences of parasite-induced phenotypic changes on host performance. Specifically, we quantified the effects of limb malformations on hosts' jumping distance, swimming ability, foraging success, and physical endurance among field-collected amphibians. We chose to use field-collected animals with naturally induced malformations to maximize the ecological realism of measured responses, given that lab-raised hosts can exhibit altered or impaired responses to relevant tasks [35]. We used a ‘competing hypotheses’ approach to compare the explanatory power of malformation status and parasite infection intensity as predictors of each aspect of host performance, thereby decoupling the direct effects of parasitism from those of parasite-induced changes in phenotype. This approach is facilitated by the fact that, in the small ponds where *Ribeiroia* often occurs, nearly all amphibian hosts are infected but there is substantial variation in malformation status and severity. We extended these results to a field-setting by comparing changes in host survival and body condition in naturally-occurring amphibians that varied in malformation status over both short-term (biweekly) and longer-term (annual) time scales. We hypothesized that malformations would reduce host performance and survival and that these patterns would be best explained by malformation status rather than infection intensity.

## Methods

### Ethics statement

All work was conducted in accordance with the policies and protocols set forth by the University of Colorado's Institutional Animal Care and Use Committee (IACUC), for which protocol number 09-07-GOO-01 was approved in May 2009.

### Study system

Our study system focuses on interactions between the trematode parasite *Ribeiroia ondatrae* (hereafter ‘*Ribeiroia’*) and its second intermediate amphibian hosts. *Ribeiroia* uses rams horn snails (Planorbidae) as its first intermediate hosts, amphibians or fishes as second intermediate hosts, and birds as definitive hosts [30], [36]. As demonstrated through both field and experimental studies, exposure of larval amphibians to *Ribeiroia* cercariae often causes reduced survival and an increased frequency of severe limb malformations, including skin webbings, bony triangles, missing limbs, and extra limbs or limb elements [27]–[28], [37]–[38].

Several factors make this study system well suited to address questions surrounding disease and the extended phenoytpe. First, *Ribeiroia* often causes malformations that are distinctive in form and severity, such that hosts with modified phenotypes can be quickly identified and collected. Limb malformations are typically rare (<3%) in most amphibian populations [28], [39], limiting the risk of abnormalities due to other factors at sites where *Ribeiroia* is abundant. Second, in ponds with moderate to high *Ribeiroia* infection intensity, a large proportion (∼30%) of emerging frogs may exhibit severe malformations, particularly in sensitive hosts such as *Pseudacris regilla* [27], [34]. This facilitates collection of large numbers of normal and abnormal individuals for use in experimental trials. Finally, in small pond systems where *Ribeiroia* is common, most if not all emerging frogs are infected (100% infection prevalence) even though not all animals are malformed, helping to decouple infection from phenotype. Moreover, while the presence and abundance of *Ribeiroia* is a strong predictor of malformations at the wetland scale [27], normal and malformed frogs from the same pond often exhibit similar levels of infection. This seemingly contradictory result stems from the fact that whether an amphibian becomes deformed depends on the exact position of invading cercariae and the timing of exposure [37]. Higher cercarial exposure increases the likelihood that some parasites disrupt limb growth, but this is primarily true if hosts are exposed during a ‘critical window’ of early limb development [32]–[33].

### Performance trials

We examined frog performance using a series of ecologically relevant locomotor performance tasks [40]–[43] that measured the burst swimming speed, jumping distance, foraging efficiency, and endurance of malformed and normal individuals. We collected 114 recently metamorphosed Pacific chorus frogs (*Pseudacris regilla*) from two ponds (Rosendin Pond, Santa Clara County and Quick Pond, Contra Costa County, California, USA) known to support both *Ribeiroia ondatrae* and a high frequency of malformed frogs [44]. These ponds are located on local parks that were historically used for livestock grazing. We used wild-caught animals rather than experimentally infected individuals to ensure performance measures, malformations types, and infection levels were representative of natural conditions. While this could introduce a potential confound in understanding the relationships among infection, phenotype, and performance, we suggest this effect will be minimal here because: (1) parasite-induced malformations affect frog limbs, which are used in locomotion only after metamorphosis, and occur after the acquisition of water-borne trematodes such as *Ribeiroia* is complete; (2) we collected frogs shortly after metamorphosis before they had extensive time to diverge in body size or foraging behavior as a function of malformation status, and (3) we necropsied a subsample (n = 60) of normal and malformed hosts to quantify *Ribeiroia* and ensure there were no differences in other forms of infection (e.g., other trematodes, soil-borne nematodes, and trophically transmitted parasites). For all trials, animals were not fed on the day that performance tests were conducted, and only one type of performance trial was conducted on a given day. We alternated trials between randomly selected normal and malformed individuals used in each performance task.

#### Swimming performance and endurance

To assess swimming performance, we placed an individual frog at one end of a polyvinyl chloride (PVC) raceway (1.5×0.12×0.1 m) filled with filtered water to a depth of 6 cm and allowed it to swim the entire length of the raceway and back again. Frogs were encouraged to swim using gentle taps by the observer's index finger and trials were halted once an individual ceased swimming following three taps by the observer. Prior to each trial, we compared the temperature of the water and the body surface of each frog using a handheld infrared thermometer (Fluke 62 Mini) to ensure no differences between groups. We timed frogs as they swam over lines placed at 10 cm intervals and estimated ‘maximum burst speed’ as the minimum time required to travel a 40 cm section on any of three replicate swims. Using similar methods, we quantified swimming endurance as the total distance (cm) and duration (s) that an individual frog swam within a circular channel (inner diameter 55 cm, outer diameter 95 cm) with a water depth of 10 cm. Lines at 10 cm intervals around the perimeter provided a measure of distance travelled during review of trial videos.

#### Jumping performance

We placed a single frog in the centre of a 2×2 m disc and encouraged it to jump by gently touching it on the back. Typically, frogs made a series of jumps in rapid succession. Following each jump, we marked the frog's landing point with a marker. We scored a total of six jumps for each frog and used the single longest jump as our measure of ‘maximum jump distance’. In cases where a frog did not make a subsequent jump, we gently touched it on the back to elicit another jump.

#### Foraging behavior

We evaluated each frog's foraging ability by adding either 5 wingless fruit flies or 1 small cricket into a 12×12×10 cm arena and quantifying time to first capture (s), time to consume a prey once captured (s), number of capture attempts (lunges), and proportion of prey items consumed. Trials lasted until all prey items were consumed or for a maximum of 300 s (fruit flies) or 720 s (cricket). Frogs, which were randomly assigned to prey type, were given a 10 min acclimation period prior to beginning trials and individual frogs were used only once.

### Field studies

#### Capture-mark-recapture (CMR) study

Every two weeks between 29 May and 5 August 2009, we monitored malformation frequency, frog abundance, and frog body condition at Sheep Pond (Contra Costa County, CA, USA), a pond with a high frequency of parasite-induced limb malformations but a small enough size (0.028 ha) to achieve a high capture efficiency of metamorphosing frogs. We captured all frogs (malformed and normal) observed along linear transects running parallel to the shoreline. Because metamorphic *P. regilla* can be captured in large numbers, regardless of malformation status, they provide an ideal system for CMR studies. Following capture, we assessed each frog's malformation status and size (snout-urostyle length, mm, and mass, g). On each sampling date except the final survey, we batch-marked frogs using biologically inert Visible Implant Fluorescent Elastomer (VIE) tags (Northwest Marine Technologies, Shaw Island, WA), which were injected into the muscle tissue of the inner thigh. This technique, which has been used successfully on a variety of amphibian species and life history stages [45]–[47], offers a significant improvement over traditional methods (e.g., toe clipping). We used a different color on each visit and normal and malformed frogs were marked on opposite legs, which were alternated between visits. Following marking, frogs were released at the edge of the pond. We calculated frog body condition by regressing frog body mass against frog size (SUL) and comparing the residuals of captured frogs for each sample week.

#### Predator exclusion experiment

As a complementary study, we compared the survival of field-collected normal and malformed frogs in two outdoor cages that excluded predators. Cages, which were built on the shoreline of the pond and contained a constant water supply, were constructed with a wooden frame and aluminum fly wire (1.0 mm mesh) covering the top and sides (0.9×0.9×1.5 m). The base of each cage was buried 0.15 m into the substrate to prevent unwanted emigration and immigration. We placed 20 normal and 20 malformed frogs into each cage, each of which were selected at random and received a fluorescent tag as described above. After one month, we dismantled the cages and assessed the survival of frogs in each cage.

#### Adult frog sampling

Finally, we revisited the pond to quantify malformation frequency in breeding adult frogs the following two springs. Because *P. regilla* generally achieve sexual maturity within 1 year [48]–[50], this is an appropriate time period over which to evaluate recruitment into the adult population. On 4 Mar 2010 and 25 Feb 2011, we captured adults at night and recorded malformation frequency and whether captured frogs were marked. Although the temporal persistence of VIE tags in frogs is not well known, we did not expect differential loss between normal and malformed frogs. On each visit, a subset of adult frogs (n = 10) were necropsied as above to measure *Ribeiroia* abundance.

### Analyses

We evaluated the effects of malformation status on frog performance using univariate ANOVAs with log_10_-transformed values of each performance response. Frog size (SUL) was included as a covariate in all analyses. To further decouple the relationships among infection, malformation status, and performance, we used a subset of the data for which we had information on all three components and (1) compared *Ribeiroia* infection abundance between normal and malformed frogs at the end of the study and (2) evaluated the relative explanatory power of a frog's infection level and its malformation status in predicting performance.

We used the program MARK [51] to assess the short-term (biweekly) survival of normal and malformed frogs in the field. We used the Cormack-Jolly-Seber (CJS) open population model, which contains two independent parameters: apparent survival (Ф) and recapture probability (*p*) [52]. Apparent survival is the estimated probability that a marked frog survives a particular time period and is recaptured at time *t*+1, while recapture probability is the likelihood that a living and marked frog is recaptured at time *t*+1, and depends on recapture data from subsequent time points (e.g., time *t*+2). Owing to this feature, recapture probability cannot be calculated independently from survival for the final time interval. Apparent survival is not considered ‘true survival’ since it does not incorporate emigration. Metamorphosing *P. regilla* typically do not emigrate away from a pond until late in the season [49]–[50], such that we considered emigration to be negligible. While new frogs continued to metamorphose over the season (birth), this should not affect recapture of marked individuals.

To analyze the influence of malformation status on survival, we used an information theoretic approach [53], in which we developed a series of a priori models that encompassed hypotheses about the effects of malformations on host survival and recapture probability. Specifically, we generated 9 univariate models that either considered survival and recapture probability to be constant, to vary with time, to vary with group (malformed vs. normal), or to vary both temporally and by group status. We were not able to include multivariate models for which individual parameters varied as a function of both time and group status owing to unreliable parameter estimates. We selected among candidate models using the small sample size adjustment to the Akaike Information Criterion (AIC_c_), and we calculated the distance of each model (ΔAIC_c_) from the model with the lowest AIC_c_ value, and the Akaike weight (*w*_i_), which is the relative likelihood of a particular model given the data [53].

## Results

### Performance trials

**Figure 1:**
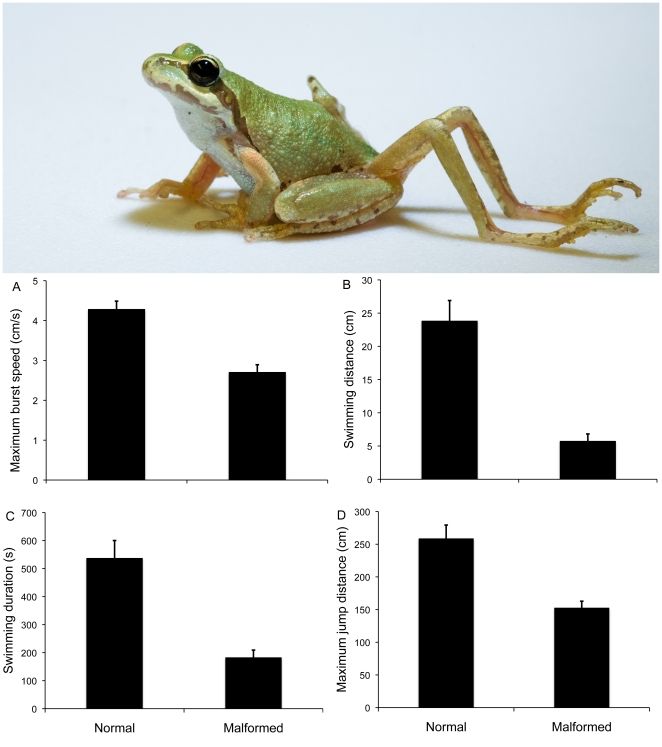
Effects of limbs malformations on the locomotory performance of Pacific chorus frogs (*P. regilla*) in laboratory trials. Above: chorus frog with parasite-induced limb malformation. Below: presented are the results of trials measuring (A) maximum burst swim speed, (B) maximum swimming distance, (C) maximum endurance time, and (D) maximum jump distance.

Of the 114 frogs used in performance trials, 63 were normal and 51 exhibited one or more malformations. Malformations affected the hind limbs and were dominated by extra limbs or feet (52.1%) and skin webbings (21.1%) (n = 71 total abnormalities) (Fig. 1). We found no differences in the initial mass or length of malformed and normal frogs (Mass: F _1, 114_ = 1.183, P = 0.279; SUL: F _1, 114_ = 0.637, P = 0.427). Among the subsample of frogs collected prior to initiating performance trials, we also found no difference in *R. ondatrae* metacercarial abundance between normal and malformed frogs (Mean infection in normal and malformed frogs = 36.2 and 42.4, respectively; t = −0.749, P = 0.461). We also found no differences in infection by other larval trematodes, including *Alaria* sp., *Manodistomum* sp. and kidney-infecting echinostomes (all P>0.05). No nematodes, cestodes, or adult trematodes were recovered (although we cannot eliminate the possibility of differences in viral, bacterial, or fungal infections).

Malformation status strongly influenced each of the measured performance responses. The mean burst swimming speed of malformed frogs was 37% slower than that of normal individuals (F _1, 113_ = 26.915, P<0.001; Fig. 1a), while their average swimming distance was 76% shorter relative to normal frogs (F _1, 66_ = 22.21, P<0.001; Fig. 1b). Similarly, swimming endurance time was 66% lower for malformed frogs (F _1, 66_ = 17.578, P<0.001; Fig. 1c). Frogs with parasite-induced malformations swam for ∼60 s relative to nearly 9 min for normal individuals (Fig. 1c). Malformed individuals also exhibited maximum jump distances that were 41% shorter than those of normal frogs (F _1, 111_ = 33.55, P = 0.006; Fig. 1d). On average ±1 SE, normal frogs jumped 258.2±21.1 cm relative to 152.3±10.6 cm for deformed frogs (Fig. 1d).

**Figure 2:**
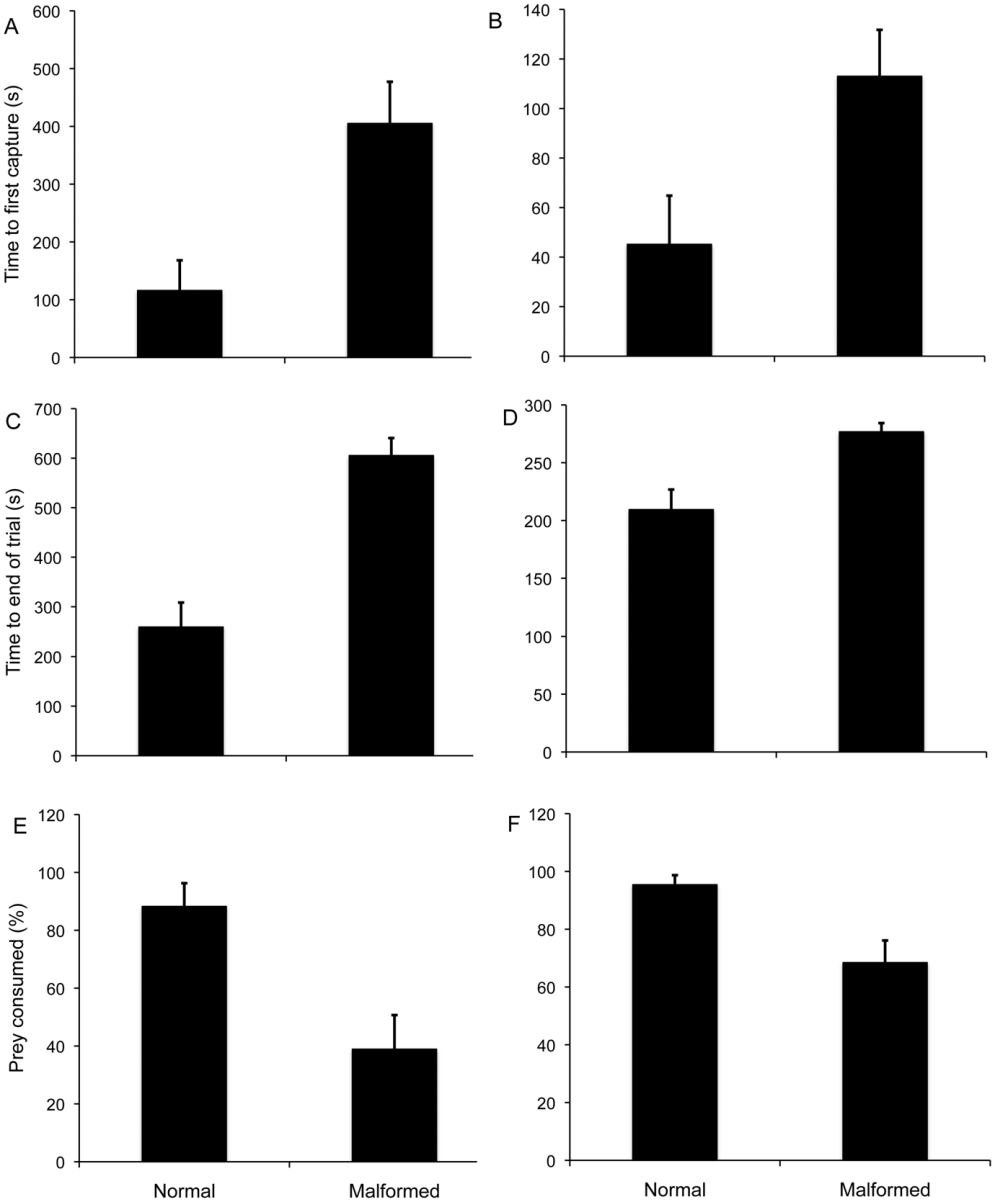
Effects of limb malformations on the prey capture ability of Pacific chorus frogs (*P. regilla*) in laboratory trials. Presented are the results of trials measuring the time required to first capture a single small cricket (A) or fruit fly (B), the time until all prey items were consumed or the trial was ended for crickets (C) or fruit flies (D), and the total proportion of prey consumed by the end of the trials for crickets (E) or fruit flies (F). Frogs were presented with either a single cricket or 5 fruit flies and allowed a maximum of 720 s and 300 s, respectively, to consume all prey before the trial was ended.

Malformed individuals were also less effective at capturing prey, requiring more time to first capture (crickets: F_1, 33_ = 18.17, n = 35; P<0.0001; fruit flies: F_1, 30_ = 12.11, n = 32; P<0.005; Figs. 2a and 2b) and more time to consume available prey items (crickets: F_1, 33_ = 33.16, n = 35; P<0.0001; fruit flies: F_1, 30_ = 16.63, n = 32; P<0.0001; Figs. 2c and 2d). Although there were no differences in the number of capture attempts made by malformed and normal frogs (crickets F_1, 33_ = 0, n = 35; P = 0.9; fruit flies: F_1, 30_ = 0.48, n = 32; P = 0.49), malformed individuals ultimately captured a lower total proportion of available prey (crickets: F_1, 33_ = 11.63, n = 35; P<0.005; fruit flies: F_1, 30_ = 7.92, n = 32; P<0.01; Figs. 2e and 2f).

After concluding performance trials, surviving frogs (n = 104) were necropsied to quantify infection by *Ribeiroia* and evaluate the explanatory influence of infection relative to malformation status. As seen with the field data, there were no significant differences in infection between malformed and normal frogs (mean infection in normal and malformed frogs = 31.2 and 36.1, respectively; t = −1.01, P = 0.316). Correspondingly, inclusion of *Ribeiroia* abundance did not increase explanatory power for any of the performance measures relative to models with malformation status alone (all P>0.05). Malformation severity, or the number of abnormalities per frog [34], correlated negatively with burst speed (ρ = −0.37, P = 0.0001), swimming distance (ρ = −0.57, P<0.0001), swimming endurance (ρ = −0.51, P<0.0001), and maximum jump distance (ρ = −0.51, P<0.0001). Among malformed frogs only (normal individuals excluded), malformation severity remained significantly correlated only with jump distance (ρ = −0.31, P = 0.035). However, most animals had 1 (65.2%) or 2 (23.9%) abnormalities, limiting the sensitivity of this approach for evaluating the influence of malformation severity on performance.

### Field Data

**Figure 3:**
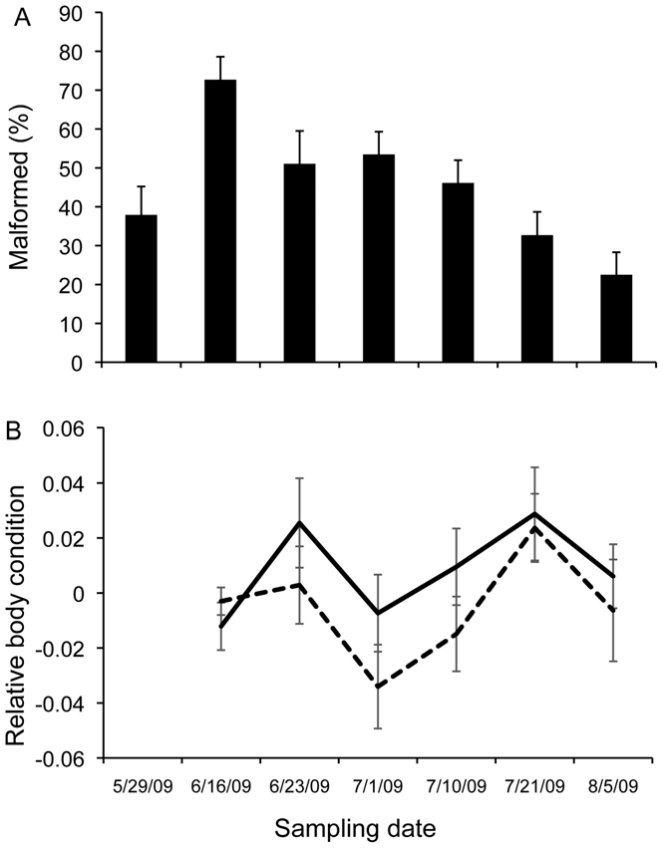
Estimates of malformation frequency and frog body condition over the summer of 2009. (A) Frequency of malformations in metamorphic chorus frogs over time. (B) Relative body condition of normal (solid line) and malformed frogs (broken line). N = 1388 sampled frogs.

**Figure 4:**
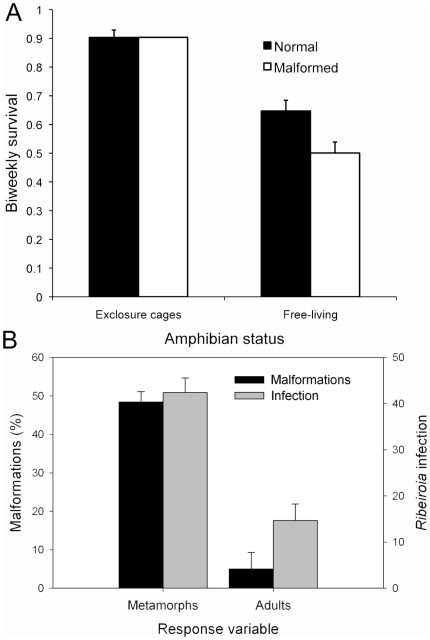
Survival, malformations, and infection data from Pacific chorus frogs (*P. regilla*) in nature. (A) Estimated biweekly survival of normal and malformed frogs as a function of whether they were free-living or maintained within predator-free exclosures. Survival estimates of free-living frogs were determined using MARK over the course of sampling events, whereas the survival of frogs in two replicate cages on the shore of a pond were estimated over 33 days and back-calculated to biweekly survival using an exponential decay function. (B) Malformation frequency and *Ribeiroia* infection intensity in metamorphic and adult frogs from Sheep Pond. Presented are the mean infection intensity + 1 SE and the overall malformation frequency with the 95% upper confidence interval for a proportion.

Between May and August 2009, we captured a total of 716 normal and 672 malformed frogs from Sheep Pond (Table S1). The frequency of malformations changed over the monitoring period (χ^2^ = 136.776, n = 1388, P<0.001), with a peak (>70%) in mid-June followed by a progressive decline (Fig. 3a). Based on AIC_c_ values, the top-ranked model from the CMR study included a time-dependent recapture rate and a group-dependent survival rate (Table 1), with malformation status negatively affecting apparent survival (β_malformed_ = −0.60 [95% CI −0.94 to −0.27]). This model was strongly supported with a model weight of 0.98, and no other models were within 8 ΔAIC_c_ (Table 1). Based on this model, biweekly survival for normal and malformed frogs was 0.64±0.038 (SE) (95% CI 0.57 to 0.72) and 0.50±0.038 (SE) (95% CI 0.43 to 0.57), respectively (Fig. 4a). Among measured frogs (n = 288), malformation status negatively predicted frog body condition (RM-ANOVA, Malformation status F_1, 1_ = 5.26, P<0.05; Time F_1, 7_ = 4.85, P<0.001; Malformation × Time: F _1, 7_ = 5.26, P = 0.545; Fig. 3b). On average, the body condition of normal frogs was ∼30× greater than that of malformed frogs. Among frogs maintained in predator-free exclosures, however, neither malformation status nor cage replicate influenced frog recovery after 33 days (χ^2^ = 0.063, P = 0.803; Fig. 4a). Of the forty frogs (20 normal and 20 malformed) placed in each cage, we recovered 16 malformed and 17 normal frogs in cage one, and 16 malformed and 15 normal frogs in cage two.

**Table 1:** Candidate models used to estimate survival probability (Ф) of normal and malformed *P. regilla* from May to July 2009.

Model ranking	Structure	AIC_c_	ΔAIC_c_	*w*_i_	Likelihood	k	Deviance
1	Ф_(g)_ p_(t)_	1357.79	0.00	0.97515	1.0000	7	108.30
2	Ф_(t)_ p_(t)_	1366.57	8.78	0.01207	0.0124	10	110.99
3	Ф_(t)_ p_(g)_	1367.25	9.46	0.00858	0.0088	7	117.77
4	Ф_(.)_ p_(t)_	1368.68	10.89	0.00420	0.0043	6	121.22
5	Ф_(t)_ p_(.)_	1384.83	27.04	0.00000	0.0000	6	137.37
6	Ф_(.)_ p_(g)_	1422.50	64.71	0.00000	0.0000	3	181.09
7	Ф_(g)_ p_(g)_	1427.35	66.68	0.00000	0.0000	4	181.05
8	Ф_(g)_ p_(.)_	1427.35	69.57	0.00000	0.0000	3	185.95
9	Ф_(.)_ p_(.)_	1435.88	78.10	0.00000	0.0000	2	196.49

Ф = survival rate, *p* = recapture rate, g = group (malformation status), t = time, AIC_c_ = sample-size adjusted Akaike Information Criterion, ΔAIC_c_ = difference between the best model and the current model, *w*_i_ = model weight, k = number of parameters, deviance = model deviance.

Despite the consistently high frequency (∼50%) of malformations in metamorphic *P. regilla* throughout the summer, we detected few malformations in adult frogs returning to breed the following springs. Of 180 adults captured in 2010 and 2011, 9 (5.0%) exhibited minor abnormalities: three supported small outgrowths, four had abnormal skin developments near the tail resorbtion site, one was missing a digit and one was missing a hand (Fig. 4b). Only two frogs captured in 2010 were marked: one normal and one abnormal. While 1 tag was intact, the second had moved within the limb. No frogs captured in 2011 were marked (n = 53 sampled). Adult frogs also exhibited significantly lower *Ribeiroia* infection intensities relative to metamorphic frogs collected the previous summer (F_1, 73_ = 8.52, P<0.005; Fig. 4b). Infections (±1 SE) in adult *P. regilla* from 2010 and 2011 averaged 21.6 (±6.8) and 6.5 (±2.9), respectively.

## Discussion

While diseases can sometimes lead to epidemic mortality, including spectacular examples such as bubonic plague in 14^th^ century humans and rinderpest in African ungulates [54]–[55], many parasites and pathogens have sublethal effects that can be difficult to isolate under natural conditions. Nevertheless, these infections can significantly alter how hosts interact with the environment, with other organisms, and even with members of their own species [e.g., 18]. When parasite-induced manipulations are beneficial to parasites [8]–[9], [56]–[58], for instance by increasing parasite growth or transmission, they can be considered realized extensions of the parasite's phenotype [2], [9], [11], [13]. In spite of growing appreciation for the role of pathogens in ecological interactions [1], [6], [59]–[60], the sublethal consequences of infections on host performance have rarely been quantified in natural systems.

Parasite-induced limb malformations in amphibians offer a valuable opportunity to investigate implications of the extended phenotype and broadly quantify the effects of disease on host attributes. Unlike many well-studied examples of parasite manipulation, which involve changes in color or behavior (see [11]), *Ribeiroia* causes fundamental changes in host body plan by altering the number and structure of hind limbs. Our results demonstrate that parasite-induced changes in phenotype deeply altered every dimension of host performance measured in this study. Malformed frogs exhibited substantial (37 to 76%) reductions in burst speed, swimming distance, endurance, and jumping distance. Correspondingly, while both groups made similar numbers of attempts, malformed frogs were less successful at capturing prey and ultimately consumed 28% and 55% fewer fruit flies and crickets, respectively. These effects were as severe or greater than those reported in similar studies undertaken with other amphibian parasites [41], [43], [61]. Because of the distinctive nature of this parasite-induced change in phenotype, for which the causal relationship with *Ribeiroia* infection has been well established [26]–[29], we avoid problems that can arise when natural variation in subtler phenotypes influences subsequent patterns of infection [23]. Furthermore, while parasitic infections often correlate negatively with host body condition, few studies have examined how such patterns translate into differences in host performance at both the individual and population-level, as done here.

Our data also provide insight into the mechanism through which phenotypic changes influence host performance. While many experimental studies compare the behavior or performance of infected and uninfected hosts, all hosts in our study were infected but varied in parasite abundance and malformation status. Based on our analyses, observed reductions in performance were driven by malformation status rather than by variation in infection intensity. On average, normal and malformed frogs supported comparable and overlapping levels of *Ribeiroia* infection, and *Ribeiroia* abundance explained no additional variation in host performance after accounting for malformation status. Malformation severity, estimated as the number of abnormalities per host, also correlated negatively with performance. This novel result reflects the inherent non-linearities in the relationship between infection and disease; the risk of pathology varies not only with parasite dosage but also with host developmental stage and the exact position of invading parasites [32]–[33], [62], such that normal and malformed frogs within the same wetland typically exhibit similar levels of infection [28]. Such ‘conditional manipulations’ can be beneficial to trophically transmitted parasites by decreasing the likelihood that the entire host population is eliminated [9], particularly for parasites with high infection prevalence, as seen here. Thus, while many examples of parasite-induced manipulations involve changes in host behavior or appearance resulting from shifts in host metabolism or physiology [8]–[11], our results indicate that the effects of *Ribeiroia* on amphibian hosts occurred through changes in body plan.

The debilitating effects of parasite-induced changes in body plan observed in the laboratory translated into reduced survival of hosts in nature. Based on a capture-mark-release study involving 1388 frogs, malformed hosts exhibited ∼22% lower biweekly survival relative to normal individuals, despite similar levels of parasite infection. Malformation status was included as a strong, negative predictor of host survival in the best-supported model. Correspondingly, field-caught frogs with one or more deformities had significantly poorer body condition relative to normal frogs. Although the specific cause of mortality in these free-living frogs is open to conjecture, both normal and malformed frogs exhibited comparably high survival in outdoor exclosures that prevented predators, suggesting that differential vulnerability to predators is likely an important contributing factor. Thus, even while abnormal hosts were significantly worse at capturing prey in experimental foraging trials, in nature they appeared to be at greater risk of predation than of starvation. In a previous field study, malformed frogs were more likely to occupy high-risk habitats and, when approached by a simulated predator, waited longer to escape and escaped over shorter distances than did normal individuals [44]. Taken together, these findings suggest that frogs with parasite-induced malformations suffer lower survival due to elevated predation.

Importantly, our results further suggest that parasite-manipulated hosts rarely recruited into the breeding adult population. While ∼50% of metamorphosing frogs at Sheep Pond exhibited malformations in 2009, <5% of returning adults in 2010 and 2011 were abnormal. The few malformations in adult frogs were minor relative to those in recently metamorphosed individuals, and adult frogs supported significantly lower levels of *Ribeiroia* infection compared with metamorphs (Fig. 4b). Considering that *P. regilla* typically reach sexual maturity within one year and live for <3 years [48]–[50], this is a reasonable time scale over which to evaluate frog survival. This suggests that, alongside larval mortality resulting directly from *Ribeiroia* infection [26], [32], [63], parasite-induced deformities reduce the survival of metamorphic individuals such that few abnormal frogs are recovered as adults (also [34]). However, given that few marked individuals were recaptured as adults, we cannot rule out the possibility that marked frogs returned to breed in different wetlands or that the loss of normal and malformed frogs was comparably high. Without longer-term data, it also remains conjectural whether malformations have population-level impacts on the viability of affected frog populations.

An important evolutionary consideration is whether parasite-induced changes in host phenotype enhance parasite persistence or transmission. According to the parasite manipulation hypothesis, a parasite-induced effect is adaptive if it 1) is complex, 2) shows signs of a purposive strategy, 3) has arisen in several lineages of hosts or parasites, and 4) increases the fitness of either the host or the parasite [9], [57]. We suggest that the *Ribeiroia*-amphibian malformation system satisfies most of these criteria. *Ribeiroia* cercariae demonstrate a remarkably non-random pattern of infection in which encystment occurs almost exclusively around the developing limb buds of larval amphibians [29], [37]. This suggests that infection in this location offers an advantage to the parasite (*purposive strategy*), although whether this is due to the resulting malformations or to other characteristics associated with this infection site (e.g., reduced hydrodynamic drag [64]) remains an open question (but see [65]). Depending on the number of infecting parasites and the developmental stage of the host, infections can lead to *complex* changes in host body plan involving the number or structure of the hind limbs. Because larval anurans depend primarily on their tails for locomotion [66], limb malformations are likely to impair host locomotion only among post-metamorphic frogs, which is also the stage expected to be vulnerable to definitive host predators (e.g., birds and mammals). Moreover, growing evidence now suggests that larval trematodes in *multiple families* induce structural malformations that likely enhance predation risk in other intermediate host taxa, including fishes [62], [67]–[68], suggesting that such developmental alterations have arisen independently in other host-parasite systems.

More challenging, however, are efforts to demonstrate conclusively that parasite-induced alterations cause increased predation (and therefore transmission) by appropriate definitive hosts rather than unsuitable predators. Indeed, owing to the logistical challenges inherent to quantifying manipulations and transmission under real-world conditions [19]–[21], [69]–[70], this criterion has rarely been demonstrated compellingly (or at all) for many of the ‘classic’ examples of parasite-induced manipulations [8]–[9], [11]. We suggest that, while these same challenges persist here, *Ribeiroia*-induced malformations will most likely increase parasite transmission because of (1) the extremely detrimental nature of the induced deformities, which impair the primary locomotory system of the host [44], (2) the broad diversity of definitive hosts suitable for *Ribeiroia*, including >50 species of birds and mammals [30], and (3) the relatively small number of consumed manipulated hosts needed to increase transmission, even when unsuitable predators also consume manipulated hosts [24], [71]. By altering foraging behavior, vulnerability to predators, and the migratory abilities of hosts, such parasite-induced changes in phenotype have the potential to influence entire ecological communities, particularly in light of the widespread distribution of *Ribeiroia* and the large number of hosts it can affect. While the current example of phenotypic manipulation is novel in its distinctiveness, we suggest that parasite-induced changes in host behavior and morphology are more widespread than often appreciated owing to their typically cryptic nature.

## Supporting Information

Table S1 Results of the capture-mark-recapture study. Listed is the number of normal and malformed individuals captured and marked, the number recaptures, the total number of captured frogs, and the percentage of individuals that were malformed.
(DOC)
