# Supplementary material for: Disease and the Extended Phenotype: Parasites Control Host Performance and Survival through Induced Changes in Body Plan
Source: PLoS One. 2011 May 25;6(5):e20193. doi: 10.1371/journal.pone.0020193 (PMC3102088; doi:10.1371/journal.pone.0020193)
Supplement: Table S1 — Results of the capture-mark-recapture study. Listed is the number of normal and malformed individuals captured and marked, the number recaptures, the total number of captured frogs, and the percentage of individuals that were malformed. (DOC) [file pone.0020193.s001.doc]

Table S1. Results of the capture-mark-recapture study. Listed is the number of normal and malformed individuals captured and marked, the number recaptures, the total number of captured frogs, and the percentage of individuals that were malformed.

| Date | Normal frogs | | Malformed frogs | | Total | Malformation (%) |
| --- | --- | --- | --- | --- | --- | --- |
|  | Captures | Recaptures | Captures | Recaptures |  |  |
| 29 May | 118 | 0 | 72 | 0 | 190 | 37.9 |
| 16 June | 56 | 3 | 149 | 1 | 205 | 72.7 |
| 23 June | 70 | 22 | 73 | 23 | 143 | 51.1 |
| 1 July | 135 | 28 | 155 | 25 | 290 | 53.5 |
| 10 July | 160 | 42 | 137 | 33 | 297 | 46.1 |
| 21 July | 177 | 46 | 86 | 24 | 263 | 32.7 |
| Total | 716 | 141 | 672 | 106 | 1388 | 48.4 |
